# A clinical scoring tool validated with machine learning for predicting severe hand–foot syndrome from sorafenib in hepatocellular carcinoma

**DOI:** 10.1007/s00280-022-04411-9

**Published:** 2022-02-28

**Authors:** Ahmad Y. Abuhelwa, Sarah Badaoui, Hoi-Yee Yuen, Ross A. McKinnon, Warit Ruanglertboon, Kiran Shankaran, Anniepreet Tuteja, Michael J. Sorich, Ashley M. Hopkins

**Affiliations:** 1 College of Medicine and Public Health, Flinders University, Adelaide, SA 5042 Australia; 2 Department of Pharmacology, Division of Health and Applied Sceinces, Prince of Songkla University, Songkhla, Thailand

**Keywords:** Sorafenib, Hepatocellular carcinoma, Hand–foot syndrome, Risk prediction

## Abstract

**Purpose:**

Sorafenib is an effective therapy for advanced hepatocellular carcinoma (HCC). Hand–foot syndrome (HFS) is a serious adverse effect associated with sorafenib therapy. This study aimed to develop an updated clinical prediction tool that allows personalized prediction of HFS following sorafenib initiation.

**Methods:**

Individual participant data from Phase III clinical trial NCT00699374 were used in Cox proportional hazard analysis of the association between pre-treatment clinicopathological data and grade ≥ 3 HFS occurring within the first 365 days of sorafenib treatment for advanced HCC. Multivariable prediction models were developed using stepwise forward inclusion and backward deletion and internally validated using a random forest machine learning approach.

**Results:**

Of 542 patients, 116 (21%) experienced grades ≥ 3 HFS. The prediction tool was optimally defined by sex (male vs female), haemoglobin (< 130 vs ≥ 130 g/L) and bilirubin (< 10 vs 10–20 vs ≥ 20 µmol/L). The prediction tool was able to discriminate subgroups with significantly different risks of grade ≥ 3 HFS (*P* ≤ 0.001). The high (score = 3 +)-, intermediate (score = 2)- and low (score = 0–1)-risk subgroups had 40%, 27% and 14% probability of developing grade ≥ 3 HFS within the first 365 days of sorafenib treatment, respectively.

**Conclusion:**

A clinical prediction tool defined by female sex, high haemoglobin and low bilirubin had high discrimination for predicting HFS risk. The tool may enable improved evaluation of personalized risks of HFS for patients with advanced HCC initiating sorafenib.

**Supplementary Information:**

The online version contains supplementary material available at 10.1007/s00280-022-04411-9.

## Introduction

Hepatocellular carcinoma (HCC) accounts for approximately 80–85% of primary liver cancers [1]. Due to the lack of symptoms in early stages, HCC is often left undetected until it progresses to advanced stages, resulting in poor prognosis [2, 3]. Sorafenib is a multi-kinase inhibitor approved for treatment of HCC; however, it is associated with multiple dose-limiting toxicities.

Dermatologic side effects are amongst the most common side effects of sorafenib [4, 5]. For example, hand–foot syndrome (HFS) of any grade is experienced by approximately 61% of patients [5]. HFS typically develops 2–4 weeks after sorafenib initiation [6] and is characterized by painful erythema, scaling, and ulceration affecting the hand and feet, which can lead to reduced patient quality of life [7, 8]. Further, the development of HFS of grade ≥ 3 is considered a serious adverse effect that may lead to dosage regimen adjustment and/or premature treatment termination [9].

Risk prediction models are used by clinicians to inform the management of patients using anti-cancer medicines [10, 11]. A clinical prediction tool for sorafenib-induced grade ≥ 2 HFS in advanced renal cell carcinoma has been previously developed [9]; however, it is unclear if this tool is generable to patients with advanced HCC where sorafenib is mostly used. Further, there is limited specificity to the prediction of sorafenib-induced grade ≥ 3 HFS, which are the events more likely to result in dose adjustments/treatment termination. This study aimed to calibrate a clinical prediction tool that allows personalized risk predictions of grade ≥ 3 HFS following sorafenib initiation for advanced HCC treatment.

## Materials and methods

### Patient population

Individual participant data (IPD) from the sorafenib arm of phase III trial NCT00699374 were used for this secondary analysis. Sorafenib was initiated at 400 mg twice daily, in 4-week cycles, to eligible participants with locally advanced or metastatic HCC [5]. Dose reduction was permitted to manage dose-limiting toxicities.

Data were made available through Project Data Sphere (www.projectdatasphere.org). Project Data Sphere is an independent non-profit, open-access cancer research platform hosting de-identified patient-level data from completed cancer clinical trials that can be shared with independent researchers to improve cancer care. Secondary analysis of anonymized IPD was exempted from review by the Southern Adelaide Local Health Network, Office for Research and Ethics as it was classified as minimal risk research.

### Predictors and outcomes

The primary assessed outcome was grade ≥ 3 HFS occurring within the first 365 days of sorafenib. Adverse effects were defined by grade according to the National Cancer Institute Common Terminology Criteria for Adverse Events (NCI CTCAE) version 3.0 [12].

Assessed pre-treatment clinicopathological variables were selected upon availability, prior evidence, and biological plausibility and included age (years), sex (male vs female), race (Asian vs Non-Asian), body mass index, ECOG performance status (ECOG PS), presence of liver/lung metastases, tumour count, leukocyte count, albumin, serum alanine aminotransferase, bilirubin, haemoglobin, estimated glomerular filtration rate, urea, and concomitant use of corticosteroids or non-steroidal anti-inflammatory drugs (NSAIDs). The rationale for selecting each of the aforementioned variables is provided in supplementary Table 1.

### Statistical analysis

A univariable Cox proportional hazard analysis was conducted to assess the association between potential predictors and grade ≥ 3 HFS. Associations were reported as hazard ratios (HR) with 95% confidence intervals (CI). Statistical significance was set at *P* < 0.05, determined via the likelihood ratio test. Continuous variables were categorised based on model fit, observed non-linearity, prior evidence, and clinically interpretable cut-points. Prediction performances were assessed via the concordance statistic (c statistic) estimated using the Harrell method [13].

A multivariable risk prediction model was developed using stepwise forward inclusion of variables with a *P* < 0.05 and the greatest improvement in the c statistic at each forward step; followed by backward deletion of variables with *P* > 0.05 and did not increase the c statistic by 0.02. The backward deletion process was conducted to find the minimal number of predictors with maintained prediction performance.

The final multivariable model was then internally validated using random forest analysis [14], a machine learning approach. Specifically, random forest analysis enabled an independent evaluation of variables of importance as compared to variables selected as important in the stepwise model. The relative importance of variables in the random forest model was determined using permutation variable importance measure as described previously [15]. The relative importance of each variable was scaled to 100, with a higher value indicating a stronger influence on predicting the outcome of interest.

The random forest was also used to assess for any signs of model overfitting. Model overfitting was assessed by training the model using the k-fold cross-validation approach (fivefold cross-valuation repeated 10 times). Using this approach, the data are split randomly into fivefold of training and test sets. The training set is used to build the model, the model is used to predict the test data, and the prediction performance of the model is recorded. This process was repeated 10 times (i.e. total number of trained models = 50) and the average (95% CI) of the prediction performance (c statistics) was reported. The values for the random forest hyperparameters were set to default and the model was trained using 1000 fitted trees.

To facilitate clinical utility, a risk prediction tool was developed from the validated final multivariable model. Whereby the estimates of the regression coefficients in the final multivariable model were scaled to the nearest integer value to allow the calculation of a linear predictor score (i.e. an interpretable risk score). The integer values allocated to each significant predictor within the final prediction model are described in the results section. The performance of the developed tool was further compared to the prediction model of grade ≥ 2 HFS proposed by Dranitsaris et al. in patients with advanced cell carcinoma receiving sorafenib [9]. Risk probabilities were assessed using the Kaplan–Meier method. Statistical analysis was performed using R version 4.1.0.

## Results

### Patient population

Data were available from 542 patients with advanced HCC who received sorafenib therapy. All patients received a starting sorafenib dose of 400 mg twice daily until disease progression or occurrence of unexpected toxicity. Median follow-up was 22 [95% CI 21–24] months in the cohort. A summary of pre-treatment patient characteristics is presented in Table 1. Of the 542 patients, 116 (21%) experienced grade ≥ 3 HFS during follow-up, with 78% of these events occurring within the first 30 days.

**Table 1:** Summary of pre-treatment patient characteristics characteristic who received sorafenib therapy for advanced hepatocellular carcinoma

Variable	Total No. 542
Hand–foot syndrome (grade 3 or more)	116 (21%)
Age (years)	
≤ 65	356 (66%)
> 65	184 (34%)
Missing	2 (< 1%)
Sex	
Female	85 (16%)
Male	457 (84%)
Race	
Asian	417 (77%)
Non-Asian	125 (23%)
Body mass index	
Normal	330 (61%)
Obese	40 (7%)
Overweight	143 (26%)
Underweight	29 (5%)
Baseline ECOG score	
0	289 (53%)
1	250 (46%)
Missing	3 (1%)
Liver metastasis	495 (91%)
Lung metastasis	211 (39%)
Tumour count (including liver)	
< 2	223 (41%)
≥ 2	319 (59%)
Bilirubin (umol/L)	
≥ 20	148 (27%)
< 10	101 (19%)
≥ 10 and < 20	289 (53%)
Missing	4 (1%)
Leukocytes (x10E9/L)	
< 10	505 (93%)
≥ 10	37 (7%)
Albumin (g/L)	
[21,37)	203 (37%)
[37,40)	124 (23%)
≥ 40	212 (39%)
Missing	3 (1%)
Alanine aminotransferase (U/L)	
< 70	427 (79%)
≥ 70	111 (20%)
Missing	4 (1%)
Estimated glomerular filtration rate	
≥ 90	300 (55%)
< 90	238 (44%)
Missing	4 (1%)
Haemoglobin (g/L)	
< 130	233 (43%)
≥ 130	309 (57%)
Corticosteroid use	15 (3%)
NSAID use	43 (8%)
Urea use	13 (2%)

Data are median (IQR) or number of patients (%)

### Prediction of grade ≥ 3 hand–foot syndrome

Univariable analysis identified bilirubin, albumin, haemoglobin, sex, race as significantly associated with the development of grade ≥ 3 HFS following sorafenib initiation (*P* < 0.05, Supplementary Table 2). On forward inclusion, bilirubin, haemoglobin, sex, race, and age were identified as the statistically significant predictors. The backwards elimination process resulted in a final multivariable model optimally defined by bilirubin (< 10 vs ≥ 10 and < 20 vs ≥ 20 µmol/L), haemoglobin (≥ 130 vs < 130 g/L), and sex (female vs male) (Table 2, *c* statistics = 0.64).

**Table 2:** Final multivariable model of grade ≥ 3 hand–foot syndrome following sorafenib initiation

	HR	95% CI	*P* value
Bilirubin (µmol/L)			< 0.001
≥ 20	1.00		
< 20 and ≥ 10	2.16	1.27–3.68	
< 10	3.12	1.71–5.68	
Haemoglobin (g/L)			< 0.001
< 130	1.00		
≥ 130	2.05	1.36–3.08	
Sex			0.005
Male	1.00		
Female	1.94	1.22–3.07	

*CI* confidence interval, *HR* hazard ratio

### Machine learning model validation

**Fig. 1:**
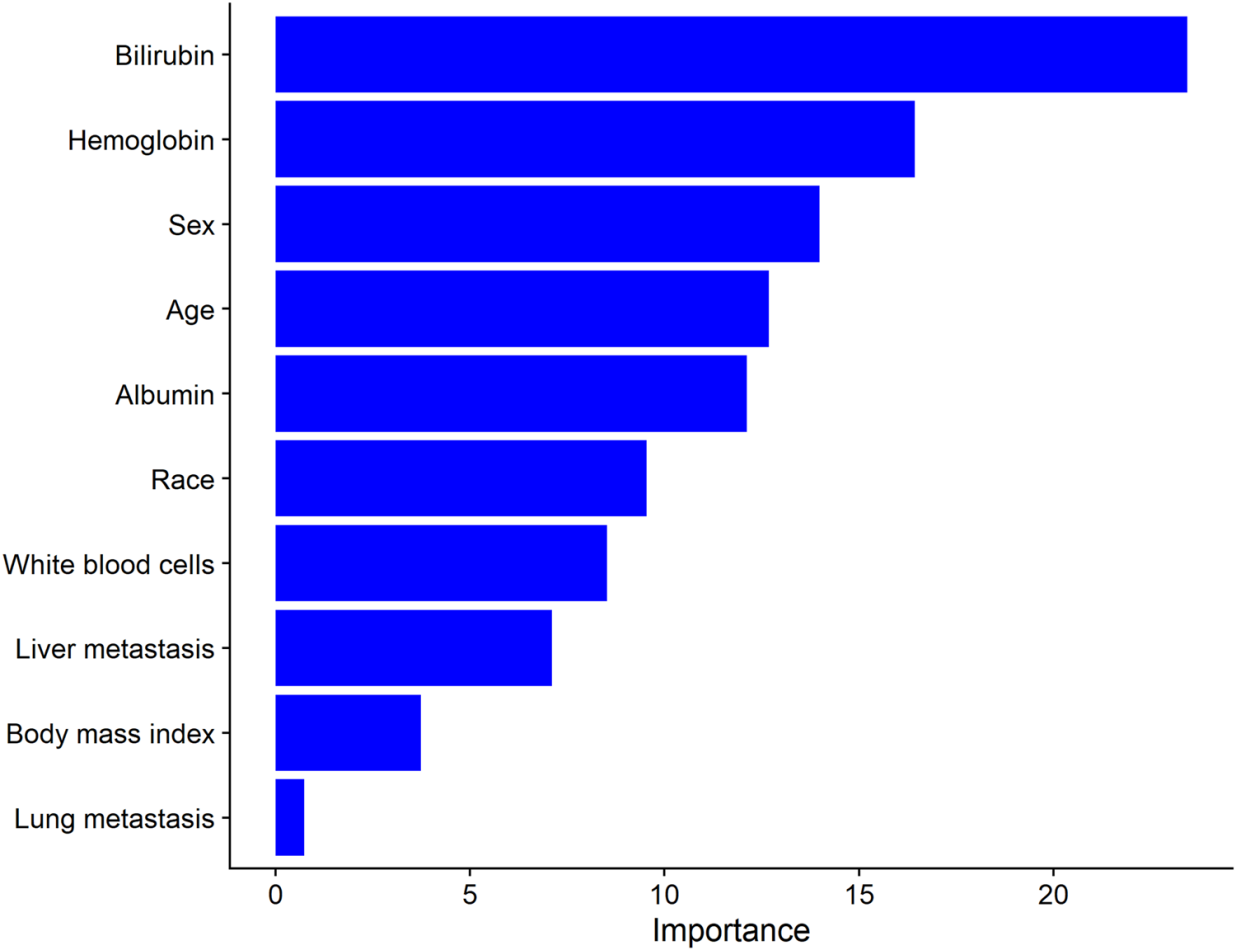
Relative importance of the top 10 variables for predicting hand–foot syndrome using random forest

The random forest machine learning model identified sex, bilirubin and haemoglobin as the most important variables in predicting grade ≥ 3 HFS confirming the validity of the variables selected in the final multivariable model. The top 10 most influential predictors of HFS from the random forest are presented in Fig. 1. The discrimination performance from the repeated cross-validated random forest model (mean, 95% CI) was 0.61 (0.59–0.63), suggesting no overfitting of the training data.

### Development of clinical prediction tool of grade ≥ 3 hand–foot syndrome

**Fig. 2:**
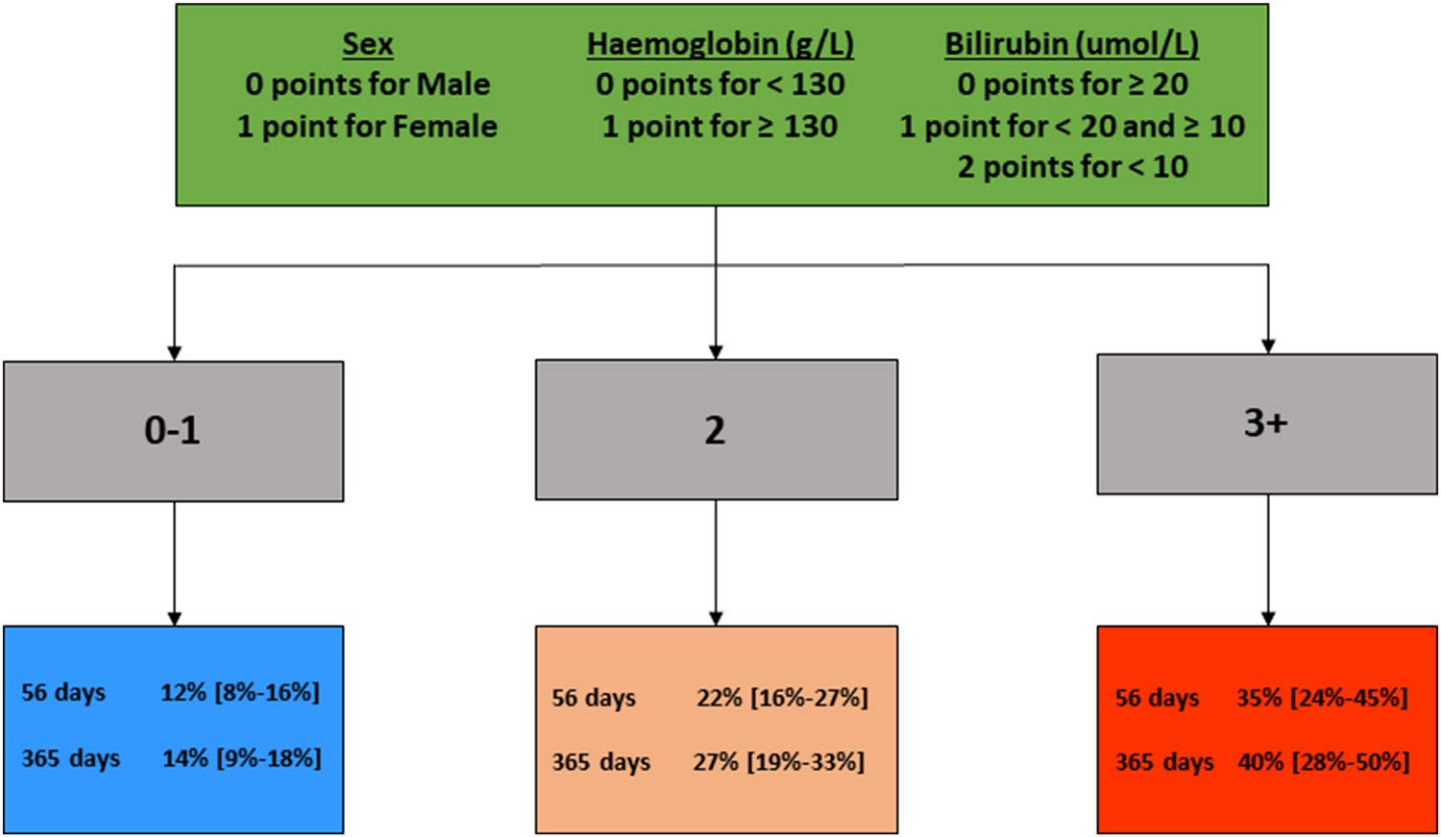
Clinical risk prediction tool of developing grade ≥ 3 hand–foot syndrome after sorafenib initiation

A risk scoring tool based on the final multivariable model was then developed. Prediction risk scores were derived from the final multivariable model where female sex equated to 1 point, haemoglobin ≥ 130 g/L equated to 1 point, bilirubin ≥ 10 and < 20 µmol/L equated to 1 point and bilirubin < 10 µmol/L equated to 2 points. Patients were then categorized into three categories (0–1, 2 and 3+) where a higher score indicates an increased risk of grade ≥ 3 HFS (Fig. 2).

The discrimination performance (mean *c*, 95%CI) of the developed risk prediction tool (Supplementary Table 3) was superior to Dranitsaris et al. risk tool for predicting grade ≥ 3 HFS (developed risk tool *c* = 0.63 [0.58–0.67], Dranitsaris et al. risk tool *c* = 0.50 [0.45–0.56], *P* < 0.001). The latter suggests that Dranitsaris et al. risk tool, which was initially developed to predict grade ≥ 2 HFS in patients with renal cell carcinoma, is not generalizable to predicting grade ≥ 3 HFS in patients with hepatocellular carcinoma.

**Fig. 3:**
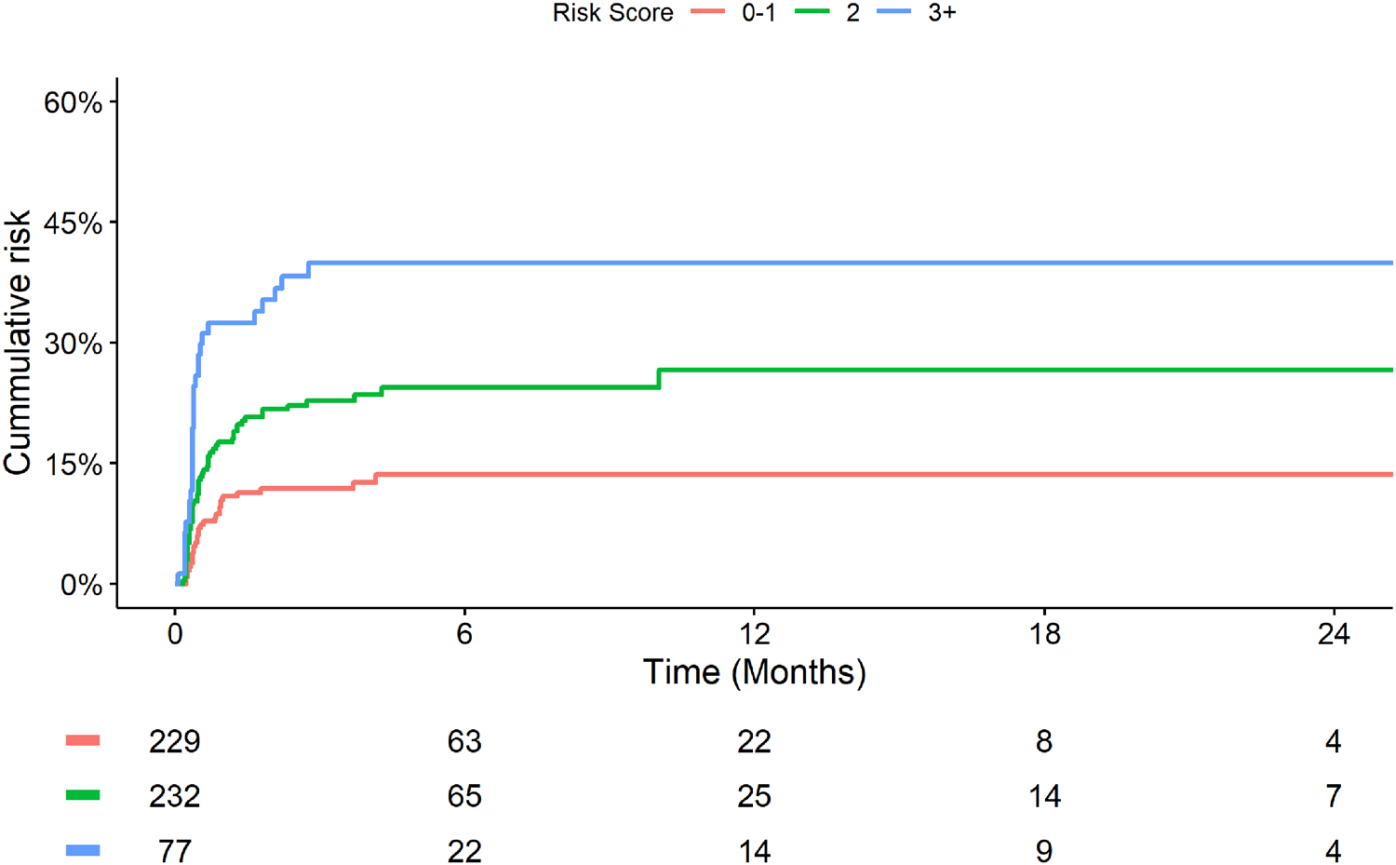
Kaplan–Meier plots of cumulative risk of grade ≥ 3 hand–foot syndrome by derived risk prediction score

The estimated probability (median, 95% CI) of developing grade ≥ 3 HFS within the first 365 days of sorafenib therapy in the high (score = 3+)-, intermediate (score = 2)- and low (score = 0–1)-risk subgroups were 40% [28–50%], 27% [19%-33%] and 14% [9–18%], respectively (Fig. 2). Figure 3 presents the Kaplan–Meier plots for grade ≥ 3 HFS according to risk score subgroups.

## Discussion

This study developed a clinical prediction tool for sorafenib-induced grade ≥ 3 HFS in advanced HCC. The model was internally validated using a random forest machine learning approach. The predicted risk of grade ≥ 3 HFS within the first 365 days of initiating sorafenib therapy ranged from 14 to 40% and was optimally defined by sex (male vs female), haemoglobin (< 130 vs ≥ 130 g/L) and bilirubin (< 10 vs ≥ 10 and < 20 vs ≥ 20 µmol/L) levels.

The female sex association with HFS following sorafenib treatment in advanced HCC is consistent with existing literature where the reported relative risk for developing HFS upon sorafenib treatment in renal cell carcinoma cohort is increased by 68% in females [9]. In addition, high baseline bilirubin was independently associated with decreased risk of HFS. Prior research assessing the association of bilirubin levels and HFS is lacking. In a small study of 83 patients, Boudou-Rouquette et al. reported an increased incidence of HFS in patients with high bilirubin levels; however, the association was confounded, and their finding was not significant upon multivariable analysis [16]. Further investigation is needed on the complex relationship between bilirubin and sorafenib-induced HFS. Further, this analysis identified high haemoglobin levels to be associated with increased risk for grade ≥ 3 HFS. A previous preliminary study identified haemoglobin as an important factor in the development of HFS [17]. While the exact mechanism is not fully elucidated, previous studies reported that sorafenib was observed to cause haemolysis of red blood cells leading to an increase in extracellular haeme [18, 19]. Extracellular haeme can cause oxidative damage and vascular injury and high-grade skin inflammation [20–23] and, therefore, can potentially contribute to the development of HFS.

The analysis presented herein demonstrates that the existing prediction model developed for predicting sorafenib-induced grade ≥ 2 HFS in advanced renal cell carcinoma population is not generalizable to patients with advanced HCC. Unlike the previous study [9], the presented analysis herein evaluated bilirubin amongst the potential predictors and was shown to be the most predictive variable for HFS in advanced HCC populations.

A strength of this analysis is the use of large high-quality data collected within clinical trial and the developed risk prediction tool. The tool being simple to use will greatly facilitate its implementation in the clinic for personalized risk predictions of grade ≥ 3 HFS in patients initiating sorafenib in advanced HCC. Being able to provide patient-specific risk predictions will enable patients and clinicians to better interpret the risk–benefit ratio of sorafenib therapy.

A potential study limitation is that the evaluation was restricted to patients treated with sorafenib within a clinical trial. Strict inclusion criteria of clinical trials may limit their generalizability to real-world populations. Therefore, external validation of the developed risk tool is necessary to assess the generalizability of the tool before the implementation of the tool for risk assessment in the clinic. Another potential limitation is the moderate prediction performance of the developed tool (*c* = 0.63) [24]. While including a broader range of predictors to build the model may improve the predictions, this analysis was limited by available sorafenib data. For example, available sorafenib data lacked information on vitamin B6/B12 and folate levels that may potentially be associated with the risk of HFS [25]. Despite these limitations, the prediction tool was able to discriminate high-, intermediate-, and low-risk patients.

In conclusion, a clinical prediction tool for sorafenib-induced grade ≥ 3 HFS was optimally developed based upon sex, haemoglobin, and bilirubin levels. The developed tool enables discrimination between risk groups and may enable improved evaluation of personalized risks of HFS for patients with advanced HCC initiating sorafenib. Future research should aim to validate this study’s findings to facilitate the transition of the development tool into clinical practice.

## Supplementary Information

Below is the link to the electronic supplementary material.

Supplementary file1 (DOCX 60 KB)

## Data Availability

Data were made available through Project Data Sphere (www.projectdatasphere.org).
