# Supplementary material for: A clinical scoring tool validated with machine learning for predicting severe hand–foot syndrome from sorafenib in hepatocellular carcinoma
Source: Cancer Chemother Pharmacol. 2022 Feb 28;89(4):479–85. doi: 10.1007/s00280-022-04411-9 (PMC8956540; doi:10.1007/s00280-022-04411-9)
Supplement: Supplementary file 1 — Supplementary file1 (DOCX 60 KB) [file 280_2022_4411_MOESM1_ESM.docx]

Supplementary Table 1. Rational for selecting pre-treatment clinicopathological variables used for the development of a grade ≥ 3 HFS prediction model

| Factor | Rationale | Reference |
| --- | --- | --- |
| Age | Age is a risk factor for developing HCC. Sorafenib safety and toxicity in elderly is of a particular concern due to comorbidities, polypharmacy, and altered pharmacokinetics of sorafenib. | [1] |
| Sex, blood cell counts, performance status, presence of lung and liver metastasis, tumour count | Prior evidence suggests that these factors were predictors for grade ≥ 2 hand-foot syndrome in patients with renal cell carcinoma treated with sorafenib. | [2] |
| Race | Preliminary evidence suggests Asian race may be associated with increased risk of developing hand-foot syndrome. | [3] |
| BMI | Prior evidence suggests BMI may be associated with increased risk of dose limiting toxicity with Sorafenib. | [4] |
| Albumin, bilirubin | Low albumin levels (Hypoalbuminemia) and high bilirubin levels indicate a compromised liver function and sorafenib is primarily metabolised by the liver and therefore interacted with drugs pharmacokinetics. Additionally, albumin and bilirubin have been assessed as predictors for survival outcomes in patients with hepatocellular carcinoma. | [5,6] |
| Alanine aminotransferase | Alanine aminotransferase is a marker for liver damage. sorafenib is primarily metabolised by the liver. |  |
| Glomerular filtration rate (GFR) | Sorafenib metabolites are excreted in the urine and therefore may be influenced by impaired renal function. Prior evidence suggests that HFS incidence was higher in patients with impaired renal function. | [7] |
| Urea | Urea has a role in prevention and lowering the severity of sorafenib-induced hand-foot syndrome. | [8] |
| Haemoglobin | Anaemia is common in cancer patients. Haemoglobin levels have been demonstrated to be an important risk factor for the development of HFS. | [9] |
| Corticosteroids | Sorafenib is primarily metabolized in the liver, by CYP3A4-mediated oxidation and UGT1A9-mediated glucuronidation. Corticosteroids (e.g. prednisolone) is a CYP3A4 inducer. Pharmacokinetic interaction between sorafenib and the prednisolone in a patient with hepatocellular carcinoma (HCC) has been previously reported. Additionally, corticosteroids have anti-inflammatory properties and are also used in the management of HFS. | [10,11] |
| Non-steroidal anti-inflammatory drugs (NSAIDs) | Prior evidence suggests that NSAID (e.g. Diclofenac) enhances oxidative stress and may potentiate sorafenib pharmacodynamic effects in patients with HCC. | [12] |

Supplementary Table 2. Univariable associations between pre-treatment characteristics and risk of sorafenib induced grade ≥ 3 hand-foot-syndrome

| **Variable** | **No. patients** | **HR** | **95% CI** | **P-value** |
| --- | --- | --- | --- | --- |
| Bilirubin (umol/L) | 538 |  |  | <0.001 |
| ≥ 20 |  | 1.00 |  |  |
| < 10 |  | 2.83 | 1.56 to 5.13 |  |
| ≥ 10 and < 20 |  | 2.16 | 1.27 to 3.67 |  |
| Albumin (g/L) | 539 |  |  | 0.014 |
| [21,37) |  | 1.00 |  |  |
| [37,40) |  | 2.00 | 1.23 to 3.24 |  |
| ≥ 40 |  | 1.60 | 1.02 to 2.50 |  |
| Hemoglobin (g/L) | 542 |  |  | 0.010 |
| < 130 |  | 1.00 |  |  |
| ≥ 130 |  | 1.67 | 1.13 to 2.47 |  |
| Race | 542 |  |  | 0.038 |
| Non-Asian |  | 1.00 |  |  |
| Asian |  | 1.68 | 1.03 to 2.75 |  |
| Baseline ECOG score | 539 |  |  | 0.067 |
| 1 |  | 1.00 |  |  |
| 0 |  | 1.42 | 0.98 to 2.07 |  |
| Sex | 542 |  |  | 0.040 |
| Male |  | 1.00 |  |  |
| Female |  | 1.59 | 1.02 to 2.47 |  |
| Body mass index | 542 |  |  | 0.251 |
| Normal |  | 1.00 |  |  |
| Obese |  | 0.95 | 0.46 to 1.98 |  |
| Overweight |  | 1.12 | 0.73 to 1.71 |  |
| Underweight |  | 2.02 | 1.06 to 3.82 |  |
| Tumour count (including liver) | 542 |  |  | 0.264 |
| <2 |  | 1.00 |  |  |
| ≥2 |  | 0.81 | 0.56 to 1.17 |  |
| Liver metastasis | 542 | 0.59 | 0.34 to 1.01 | 0.054 |
| Age (years) | 540 |  |  | 0.219 |
| ≤65 |  | 1.00 |  |  |
| >65 |  | 1.26 | 0.87 to 1.84 |  |
| Alanine aminotransferase (U/L) | 538 |  |  | 0.122 |
| < 70 |  | 1.00 |  |  |
| ≥ 70 |  | 0.67 | 0.40 to 1.11 |  |
| Leukocytes (x10E9/L) | 542 |  |  | 0.236 |
| < 10 |  | 1.00 |  |  |
| ≥ 10 |  | 0.58 | 0.24 to 1.43 |  |
| Lung metastasis | 542 | 1.08 | 0.75 to 1.57 | 0.673 |
| NSAID use | 542 | 1.23 | 0.66 to 2.29 | 0.516 |
| Estimated glomerular filtration rate | 538 |  |  | 0.923 |
| ≥90 |  | 1.00 |  |  |
| <90 |  | 0.98 | 0.68 to 1.42 |  |
| Urea use | 542 | 0.72 | 0.18 to 2.91 | 0.645 |
| Corticosteriod use | 542 | 0.93 | 0.30 to 2.93 | 0.902 |
| CI=confidence interval, ECOG=Eastern Cooperative Oncology Group, HR=hazard ratio, NSAID=non-steroidal anti-inflammatory drugs | | | | |

Supplementary Table 3. Cox proportional analysis of model-derived risk scores for development of grade ≥ 3 hand-foot syndrome

|  | **N** | **HR** | **95% CI** | **P-value** |
| --- | --- | --- | --- | --- |
| Risk Score |  |  |  | <0.001 |
| 0-1 | 229 | 1.00 |  |  |
| 2 | 232 | 1.97 | 1.26 to 3.10 |  |
| 3+ | 77 | 3.73 | 2.24 to 6.19 |  |
| CI=confidence interval, HR=hazard ratio | | | | |

# References

1. Hajiev S, Allara E, Motedayеn Aval L, Arizumi T, Bettinger D, Pirisi M, Rimassa L, Pressiani T, Personeni N, Giordano L, Kudo M, Thimme R, Park JW, Taddei TH, Kaplan DE, Ramaswami R, Pinato DJ, Sharma R (2021) Impact of age on sorafenib outcomes in hepatocellular carcinoma: an international cohort study. Br J Cancer 124 (2):407-413. doi:10.1038/s41416-020-01116-9

2. Dranitsaris G, Vincent MD, Yu J, Huang L, Fang F, Lacouture ME (2012) Development and validation of a prediction index for hand-foot skin reaction in cancer patients receiving sorafenib. Annals of oncology : official journal of the European Society for Medical Oncology 23 (8):2103-2108. doi:10.1093/annonc/mdr580

3. Chanprapaph K, Rutnin S, Vachiramon V (2016) Multikinase Inhibitor-Induced Hand-Foot Skin Reaction: A Review of Clinical Presentation, Pathogenesis, and Management. American journal of clinical dermatology 17 (4):387-402. doi:10.1007/s40257-016-0197-1

4. Antoun S, Baracos VE, Birdsell L, Escudier B, Sawyer MB (2010) Low body mass index and sarcopenia associated with dose-limiting toxicity of sorafenib in patients with renal cell carcinoma. Annals of oncology : official journal of the European Society for Medical Oncology 21 (8):1594-1598. doi:10.1093/annonc/mdp605

5. Johnson PJ, Berhane S, Kagebayashi C, Satomura S, Teng M, Reeves HL, O'Beirne J, Fox R, Skowronska A, Palmer D, Yeo W, Mo F, Lai P, Iñarrairaegui M, Chan SL, Sangro B, Miksad R, Tada T, Kumada T, Toyoda H (2015) Assessment of liver function in patients with hepatocellular carcinoma: a new evidence-based approach-the ALBI grade. J Clin Oncol 33 (6):550-558. doi:10.1200/jco.2014.57.9151

6. Hansmann J, Evers MJ, Bui JT, Lokken RP, Lipnik AJ, Gaba RC, Ray CE, Jr. (2017) Albumin-Bilirubin and Platelet-Albumin-Bilirubin Grades Accurately Predict Overall Survival in High-Risk Patients Undergoing Conventional Transarterial Chemoembolization for Hepatocellular Carcinoma. Journal of vascular and interventional radiology : JVIR 28 (9):1224-1231.e1222. doi:10.1016/j.jvir.2017.05.020

7. Parsa V, Heilbrun L, Smith D, Sethi A, Vaishampayan U (2009) Safety and efficacy of sorafenib therapy in patients with metastatic kidney cancer with impaired renal function. Clinical genitourinary cancer 7 (2):E10-15. doi:10.3816/CGC.2009.n.015

8. Lee YS, Jung YK, Kim JH, Cho SB, Kim DY, Kim MY, Kim HJ, Seo YS, Yoon KT, Hong YM, Lee JH, Lee HW, Yim HJ, Jang BK, Jang ES, Jang JY, Hwang SY (2020) Effect of urea cream on sorafenib-associated hand-foot skin reaction in patients with hepatocellular carcinoma: A multicenter, randomised, double-blind controlled study. European journal of cancer (Oxford, England : 1990) 140:19-27. doi:10.1016/j.ejca.2020.09.012

9. Naito M, Yamamoto T, Hara S, Shimamoto C, Miwa Y (2017) Hemoglobin Value Is the Most Important Factor in the Development of Hand-Foot Syndrome under the Capecitabine Regimen. Chemotherapy 62 (1):23-29. doi:10.1159/000445866

10. Noda S, Shioya M, Hira D, Fujiyama Y, Morita SY, Terada T (2013) Pharmacokinetic interaction between sorafenib and prednisolone in a patient with hepatocellular carcinoma. Cancer chemotherapy and pharmacology 72 (1):269-272. doi:10.1007/s00280-013-2187-9

11. Brose MS, Frenette CT, Keefe SM, Stein SM (2014) Management of sorafenib-related adverse events: a clinician's perspective. Seminars in oncology 41 Suppl 2:S1-s16. doi:10.1053/j.seminoncol.2014.01.001

12. Duval AP, Troquier L, de Souza Silva O, Demartines N, Dormond O (2019) Diclofenac Potentiates Sorafenib-Based Treatments of Hepatocellular Carcinoma by Enhancing Oxidative Stress. Cancers 11 (10). doi:10.3390/cancers11101453
